# Heart rate variability during sleep stages in older adults with mild cognitive impairment

**DOI:** 10.1002/alz70856_097645

**Published:** 2025-12-24

**Authors:** Alejandra Rosales‐Lagarde, Erik Leonardo Mateos‐Salgado, Ramiro Domínguez‐Aguilar, Lourdes Cubero‐Rego, Erika E. Rodríguez‐Torres

**Affiliations:** ^1^ Secretaría de Ciencia, Humanidades, Tecnología e Innovación, Mexico City, DF, Mexico; ^2^ Universidad Latina, Mexico City, DF, Mexico; ^3^ Instituto Nacional de Psiquiatría Ramón de la Fuente Muñiz, Mexico City, DF, Mexico; ^4^ Facultad de Psicología/Universidad Nacional Autónoma de México, Mexico City, DF, Mexico; ^5^ Universidad Autónoma del Estado de Hidalgo, Mineral de la Reforma, HG, Mexico; ^6^ Universidad Nacional Autónoma de México, Querétaro, QA, Mexico; ^7^ Academic Area of ​​Mathematics and Physics, Autonomous University of the State of Hidalgo, Mineral de la Reforma, HG, Mexico

## Abstract

**Background:**

Several dementias are associated to cardiovascular disease risk factors. Mild cognitive impairment (MCI) can lead to dementia. One of the less known possible cardiovascular risk factor comprises a lower index of arousals per hour during Rapid Eye Movement (REM) sleep. Therefore, the aim of this pilot study was to compare heart rate variability (HRV) during sleep stages in people with MCI and those without it considering two methodologies: spectral and Markov chain analysis.

**Method:**

Seven older adults with MCI (68.6 ± 2 years old) and the same number of older adults without it (64.6 ± 4 years old) underwent a one‐night polysomnography. Sleep stages were classified and Interbeat Intervals (IBI) were obtained from the electrocardiogram during each sleep stage. From the spectral analysis, two frequencies were considered, low (LF) and high frequency (HF). A Markov chain analysis was calculated between probabilities of transitions from arrhythmic to rhythmic states during each sleep stage.

**Result:**

Between both groups, there were not statistically significant differences in LF and HF spectral analyses within sleep stages. Instead, in the Markov chain analyses, probabilities of transitions from arrhythmic to rhythmic states during N2 and REM sleep were significant between groups.

**Conclusion:**

Further studies are needed to disentangle the relationships between transitions of the HRV and the lower index of arousals per hour during REM sleep in MCI. Older adults with MCI having fewer arousals per hour in REM sleep also could result to be more arrhythmic than controls.

